# Cerebrospinal fluid neurofilament light chain levels in children with acquired demyelinating syndrome

**DOI:** 10.3389/fped.2024.1467020

**Published:** 2024-11-05

**Authors:** Wenlin Wu, Chi Hou, Wenxiao Wu, Huiling Shen, Yiru Zeng, Lianfeng Chen, Yinting Liao, Haixia Zhu, Yang Tian, Bingwei Peng, Wen-Xiong Chen, Xiaojing Li

**Affiliations:** Department of Neurology, Guangzhou Women and Children’s Medical Center, Guangzhou Medical University, Guangzhou, Guangdong, China

**Keywords:** acquired demyelinating syndrome, ADEM, MOGAD, NMOSD, neurofilament, biomarker, children

## Abstract

**Objective:**

To study the cerebrospinal fluid (CSF) neurofilament light chain (NfL) in pediatric acquired demyelinating syndrome (ADS) and its association with factors of laboratory and imaging results.

**Methods:**

We analyzed clinical data from children with ADS collected from May 2020 to January 2021 at the Department of Neurology of Guangzhou Women and Children's Medical Center. Enzyme-linked immunosorbent assays were used to detect the CSF NfL of patients.

**Results:**

Thirty pediatric ADS patients (17 male, 13 female) were included in the study. The most frequent diagnosis was uncategorized ADS (36.7%, 11/30), followed by acute disseminating encephalomyelitis (ADEM) (23.3%, 7/30), myelin oligodendrocyte glycoprotein antibody-associated disorder (MOGAD) (20.0%, 6/30), NMO (6.7%, 2/30), multiple sclerosis (MS) (6.7%, 2/30), and neuromyelitis optic spectrum disorders (NMOSD) (6.7%, 2/30). The median CSF NfL for the first time was 7,425.28 pg/ml (interquartile range, 1,273.51, >10,000 pg/ml). CSF NfL increase over normal value (<290.00 pg/ml for people younger than 30 years old) was seen in 98.7% of patients. Patients were divided into uncategorized ADS, ADEM, MOGAD, and MS/NMO/NMOSD groups, with no significant difference in CSF NfL between each group. The CSF NfL positively correlated with the immunoglobulin (Ig) G (*ρ* = 0.473) and IgE (*ρ* = 0.366). However, the CSF NfL did not correlate with CSF white blood count and CSF protein. Furthermore, there was no significant difference between patients with oligoclonal bands positive and without. The CSF NfL negatively correlated with interferon *γ* (*ρ* = −0.501), CD45 ^+^ CD3^+^ T (*ρ* = −0.466), CD45 ^+^ CD3 ^+^ CD4^+^ T (*ρ* = −0.466), and CD45 ^+^ CD3 ^+^ CD8^+^ T cells (*ρ* = −0.521). However, it did not correlate with CD45 ^+^ CD19^+^ B cells. CSF NfL in patients with cerebral white matter lesions in MRI was higher than in patients without. Moreover, the CSF NfL positively correlated with the number of brain MRI locations (*ρ* = 0.362). Nine patients underwent multiple detections of CSF NfL, and their CSF NfL for the last detection was not significantly different from the first.

**Conclusions:**

The CSF NfL increases significantly in pediatric ADS, and it can be a biomarker of neuro-axonal injury and a good indication of the extent of lesions.

## 1 Introduction

Acquired demyelinating syndrome (ADS) comprises a spectrum of monophasic and recurrent inflammatory conditions of the central nervous system (CNS), such as acute disseminating encephalomyelitis (ADEM), neuromyelitis optic spectrum disorders (NMOSD), multiple sclerosis (MS), and clinically isolated syndromes, myelin oligodendrocyte glycoprotein antibody-associated disorder (MOGAD) (1). In young children, ADEM is the most common manifestation of ADS, and during adolescence, other ADS become increasingly common, such as MS and clinically isolated syndromes (2). Myelin oligodendrocyte glycoprotein (MOG) is on the myelin surface, acting as a cellular adhesive molecule to regulate the stability of the oligodendrocyte microtubule (3). Antibodies against MOG have been associated with various spectrum diseases, called MOGAD, including acquired demyelinating syndromes such as optic neuritis (ON), myelitis, optic neuromyelitis (ONM), ADEM, NMOSD, or MS. MOG-IgG is detected in up to 40% of pediatric patients with ADS (4).

In mature myelinated axions, the single most abundant protein is neurofilaments which are neuronal-specific heteropolymers conventionally consisting of a triple of light [neurofilament light (NFL)], medium (NfM), and heavy (NfH) chains according to molecular mass (5). After neuro-axonal damage, NfL is released into the cerebrospinal fluid (CSF) and serum (6). CSF NfL is a biomarker in several diseases characterized by axonal loss, including stroke, small vessel disease, head injury, amyotrophic lateral sclerosis, Alzheimer's disease, Huntington's disease, acute spinal cord injury, NMOSD, and MS (7). Moreover, NfL also has potential applications in systemic or neurological infectious diseases. Blood NfL levels were elevated in the acute phase of COVID-19 patients without major central nervous system manifestations and were associated with clinical severity and poor outcomes (8). However, the lack of reliable biomarkers is a big unmet need in pediatric ADS and the study about the CSF NfL in pediatric ADS is less (9). In this study, we aimed to investigate the CSF NfL in pediatric ADS and its association with factors of laboratory and imaging results.

## 2 Subjects and methods

### 2.1 Subjects

Children with ADS who were treated from May 2020 to January 2021 at the Department of Neurology of Guangzhou Women and Children's Medical Center were included. This study was approved by the Ethics Committee of Guangzhou Women and Children Medical Center (Approval No.: [2019]40701), and written informed consent was obtained from individual or guardian participants. In addition, clinical data, including demographic data, prodromal events, clinical manifestations, laboratory investigations, brain MRI, treatment, outcomes, and prognosis, were retrospectively reviewed.

The inclusion criteria were as follows: (1) age at onset < 18 years; (2) with an acute event suggestive immune-mediated central nervous system (CNS) demyelinating disorder not attributable to other conditions (infectious, metabolic, neoplastic, congenital, or vascular illness); and (3) undergoing CSF NfL detection. The diagnostic criteria for MOGAD, proposed by Jarius S et al., are as follows: (1) monophasic or relapsing acute ON, myelitis, brainstem encephalitis, or encephalitis or any combination of these syndromes; (2) MRI or electrophysiological (visual evoked potentials in patients with isolated ON) findings compatible with CNS demyelination; and (3) seropositivity for MOG-IgG as detected using a cell-based assay employing full-length human MOG as target antigen (10). ADEM is diagnosed according to the criteria proposed by the International Pediatric MS Study Group (IPMSSG) 2013 (11). Acquired demyelinating syndromes are classified according to IPMSSG criteria (11), but MS and NMOSD followed with more recent criteria (12, 13). An uncategorized diagnosis of ADS is defined as any immune-mediated CNS demyelinating disorder including clinically isolated syndrome, myelitis, and ON that does not fall into the abovementioned categories. Relapses were defined as the development of new neurological symptoms 1 month after the onset of the initial attack or, in the case of ADEM, 3 months after the onset of the initial attack (14). Medical records were independently reviewed by three neurologists for study inclusion and clinical data abstraction. The diagnosis of each case was discussed in a consensus meeting attended by at least three neurologists, and any discrepancies were resolved through discussion until a unanimous decision was reached. When dividing patients according to the diagnosis, uncategorized ADS, ADEM, and MS/NMO/NMOSD groups do not include patients with MOGAD, although they present such clinical phenotype. Disease severity is assessed using the Expanded Disability Status Scale (EDSS).

### 2.2 Methods

#### 2.2.1 Antibodies and CSF NfL test

MOG-IgG and AQP4 IgG in serum were detected by the fixed cell-based assay commercial kit (Shaanxi Maiyuan Biotechnology Co., Ltd, Shanxi, China), and these methods were reported in detail in our previous study (15, 16). Two milliliters of CSF were collected for NfL detection before the treatment by lumbar puncture in all cases and stored at −80℃. The first sample for all cases was taken at the onset or relapse attack within 1 week of either the initial onset or a relapse episode. Subsequent samples were collected during the clinical remission period or, in the case of a relapse, within 1 week of the relapse onset, subject to parental or guardian consent. The CSF NfL was detected by enzyme-linked immunosorbent assays [NFL: NF-Light kit (ELISA), catalog number 10–7001; UmanDiagnostics, Umeå, Sweden].

#### 2.2.2 The serum and CSF cytokine test

For CSF cytokine analysis, the CSF (3–4 ml) was centrifuged at 3,000 rpm for 5 min to remove white blood cells, and supernatants were stored at −80℃ until the analysis of cytokines. The levels of cytokines including tumor necrosis factor β, interleukin (IL) 12p70, IL-1 β, IL-10, IL-6, tumor necrosis factor α, interferon γ (IFN-γ), IL-8, IL-4, IL-5, IL-17A, and IL-22 in both serum and CSF were measured by the BD FACSCanto II flow cytometer according to the flow immunofluorescence method through Aimplex cytokine detection kit (QuantoBio, Beijing, China). Serum and CSF cytokine analysis was performed by the laboratory department in our hospital. These methods had been reported in detail in their previous study (17).

#### 2.2.3 Analysis of peripheral blood lymphocyte subsets

Peripheral blood lymphocyte subsets including CD45 ^+^ CD3^+^ T cells, CD45 ^+^ CD3 ^+^ CD4^+^ T cells, CD45 ^+^ CD3 ^+^ CD 8^+^ T cells, and CD45 ^+^ CD19^+^ B cells were tested with flow cytometry (BD Multitest IMK kit, catalog number 340403; BD Biosciences, CA, USA). Peripheral blood lymphocyte subsets test was performed by the laboratory department in our hospital. These methods had been reported in detail in their previous study (18).

#### 2.2.4 Assessment of brain and spinal cord lesion location and number on MRI

Brain and spinal MRI images were obtained in all patients by using 3.0 Tesla MRI Siemens. Typical brain MRI sequences included sagittal T1, T2, axial T1, T2, coronal T1, T2, short-tau inversion recovery, and fluid-attenuated inversion recovery T2, with gadolinium administered. For spinal MRI, typical sequences included sagittal T1, T2, axial T1, T2, short-tau inversion recovery, and fluid-attenuated inversion recovery T2, with gadolinium administered. MRI lesions and their location were evaluated by two independent radiologists, and the distribution of MRI lesions was categorized into the following locations: cortical gray matter, cerebral white matter, optic nerve, basal ganglia, diencephalon, cerebellum, brainstem, and spinal cord. The total number of lesions was calculated as the sum of those found in these locations. A consensus meeting, attended by at least two radiologists, was held for each case, and any discrepancies were resolved through discussion until a unanimous decision was reached.

#### 2.2.5 Statistical analysis

Statistical analysis was performed using SPSS IBM 20.0. Quantitative data with normal distribution were described by mean ± SD, otherwise median with the interquartile range (IQR). Qualitative data were described by frequency and percentage. Pearson’s Chi-square test or Fisher’s exact test was used to compare the gender ratio between males and females. An independent *t*-test or one-way ANOVA analysis [with *post hoc* testing using the least significant difference (LSD) test] was used for comparisons of age. The Mann–Whitney *U* or Kruskal–Wallis *H* test was applied for comparisons of CSF NfL levels. The correlation between CSF NfL levels and variables such as age, laboratory results, EDSS, and the number of MRI lesions was assessed using Spearman’s correlation test. A *p*-value of <0.05 (two-sided) was considered significant. Figures were graphed using GraphPad Prism 7.01 (GraphPad Software, Inc., USA).

## 3 Results

### 3.1 Demographic features and NfL in different diagnosis

Thirty pediatric ADS patients (17 male, 13 female) were included. The onset age was 8.2 ± 2.8 years. The most frequent diagnosis seen was uncategorized ADS (36.7%, 11/30), followed by ADEM (23.3%, 7/30), MOGAD (20.0%, 6/30), NMO (6.7%, 2/30), MS (6.7%, 2/30), and NMOSD (6.7%, 2/30). In 11 uncategorized ADS patients, 8 patients were diagnosed with clinically isolated syndrome, 2 with myelitis, and 1 with ON. In six MOGAD patients, two patients were diagnosed with ADEM, two with NMO, and two with ADS with anti-MOG positive. Two NMOSD patients were anti-AQP4 positive (detailed demographic data are presented in Table 1).

**Table 1:** Demographic, clinical, and CSF NfL data of patients.

	First detection at onset (*n* = 21)	First detection at relapse attack (*n* = 9)	*P*	Uncategorized ADS	ADEM	MOGAD	MS/NMO/NMOSD	*P*
Onset Age (mean ± SD)	8.5 ± 2.9	7.3 ± 2.6	0.296^a^	6.7 ± 2.0	9.3 ± 3.5	10.2 ± 2.1	7.3 ± 2.5	0.042^c,d,g^
Male:female	12:9	5:4	1.000^e^	4:7	6:1	3:3	4:2	0.202^e^
CSF NfL (pg/ml)	7,893.05 (IQR 2,985.93, >10,000)	2,668.6 (IQR 888.0, >10,000)	0.237^b^	4,573.39 (IQR 885.65, >10,000)	>10,000 (IQR 7,893.05, >10,000)	6,443.53 (IQR 1,908.575, >10,000)	5,996.82 (IQR 907.17, >10,000)	0.451^f^

ADS, acquired demyelinating syndrome; ADEM, acute disseminating encephalomyelitis; AQP4, aquaporin-4; CSF, cerebrospinal fluid; IQR, interquartile range; MOGAD, myelin oligodendrocyte glycoprotein antibody-associated disorder; MS, multiple sclerosis; NfL, neurofilament light; NMOSD, neuromyelitis optic spectrum disorders; ON, optic neuritis; ONM, optic neuromyelitis.

^a^ Independent *t*-test.

^b^ Mann–Whitney *U* test.

^c^ One-way ANOVA analysis.

^d^ LSD test.

^e^ Fisher’s exact test.

^f^ Kruskal–Wallis test.

^g^ The onset age of patients with uncategorized ADS was younger than patients with ADEM and MS/NMO/NMOSD.

**Figure 1:**
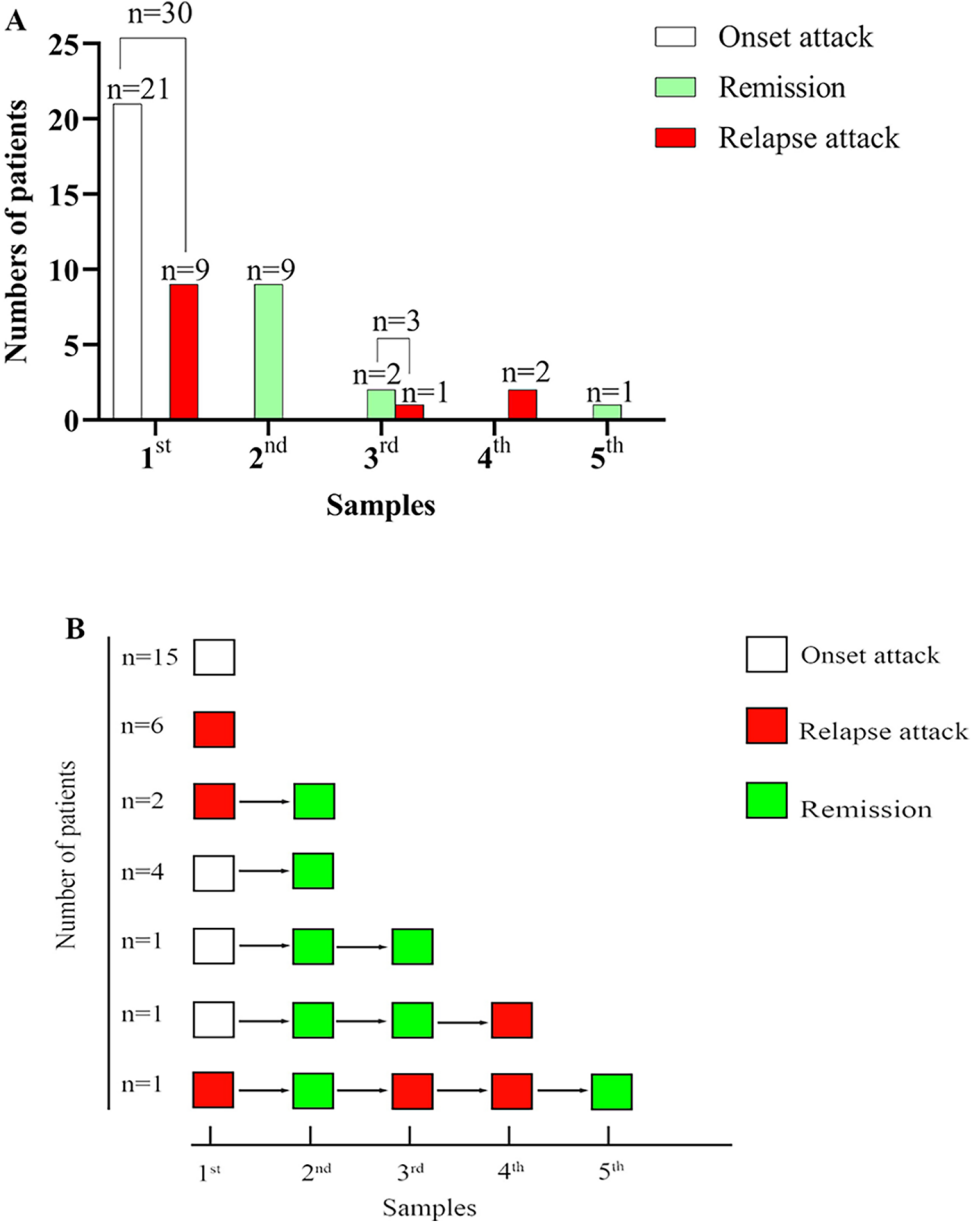
The number of patients, sample size, and disease course at different sampling. **(A)** Summary of the number of patients, sample size, and disease course at different sampling. **(B)** The disease course at different sampling for every patient.

In total, 45 samples from 30 patients were included. Twenty-one samples were taken at onset, 12 samples were taken at relapse attack, and 12 samples were taken in remission at follow-up. In 21 patients, only one sample was included, with 15 samples taken at onset and 6 at relapse attack. In contrast, multiple samples were available in the others (five in one case, four in one case, three in one case, and two in six cases) (more details are shown in Figure 1).

The median CSF NfL for the first sample in all patients was 7,425.28 pg/ml (IQR 1273.51, >10,000 pg/ml). CSF NfL increase over normal value (<290.00 pg/ml for people younger than 30 years old) was seen in 98.7% (29/30) of patients. In patients who underwent the first CSF NfL test, the median EDSS was 4.5 (IQR 3.0, 8.0). CSF NfL positively correlated with EDSS (Spearman's rank correlation coefficient, *ρ* = 0.721, *P* < 0.001). There was no significant difference in CSF NfL between girls and boys (Mann–Whitney *U* test, *Z* = −1.094, *P* = 0.274). Moreover, the CSF NfL did not correlate with age (Spearman's rank correlation coefficient, *ρ* = 0.278, *P* = 0.137). A total of 21 patients underwent their first CSF NfL examination at disease onset. Their median EDSS was 5.0 (IQR 3.0, 8.5), and the median CSF NfL level was 7,893.05 pg/ml (IQR 2,985.93, >10,000 pg/ml), which was elevated beyond the normal range in 95.2% (20/21) of patients. CSF NfL levels were positively correlated with EDSS (Spearman's rank correlation coefficient, *ρ* = 0.809, *P* < 0.001). All 21 patients received intravenous methylprednisolone (IVMP) with a dose of 15–30 mg/kg/d for 3–5 days tailed to oral prednisone and intravenous immunoglobulin (IVIG) administered at 2 g/kg over 2–3 days. Nine patients had their first CSF NfL examination during a relapse attack, with a median CSF NfL level of 2,668.6 pg/ml (IQR 888.0, >10,000 pg/ml) and a median EDSS of 5.0 (IQR 3.0, 8.5), both exceeding normal values. In this group, CSF NfL levels did not correlate with EDSS (Spearman's rank correlation coefficient, *ρ* = 0.420, *P* = 0.261). Among the nine patients who had their first CSF NfL examination during a relapse, five were diagnosed with MOGAD, two with MS, and two with NMOSD. Of the five MOGAD patients, three received rituximab (RTX) for maintenance therapy, one was treated with mycophenolate, and one received a low dose of oral prednisone as maintenance immunotherapy (initial dose was 2 mg/kg/d and reduced to 2.5–5 mg/d every 2 weeks until 5 mg/d, 5 mg/d for 1–2 months and reduced to 2.5 mg/d for 2 months, and then 2.5 mg every other day for 2 months) for about 11 months. Among the two MS patients, one received RTX as disease-modifying therapy, while the other did not receive any due to parental refusal. Both NMOSD patients were treated with RTX for maintenance therapy. There was no significant difference in CSF NfL levels between patients at onset and those at relapse attacks (Mann–Whitney *U* test, Z = −1.183, *P* = 0.237). We analyzed the association between the CSF NfL for the first sample with patients’ diagnoses. We divided patients into uncategorized ADS, ADEM, MOGAD, and MS/NMO/NMOSD groups, and there was no significant difference in CSF NfL between each group (Kruskal–Wallis test, *χ*^2^ = 0.451, *P* = 0.451).

### 3.2 The association between NfL and laboratory results

**Figure 2:**
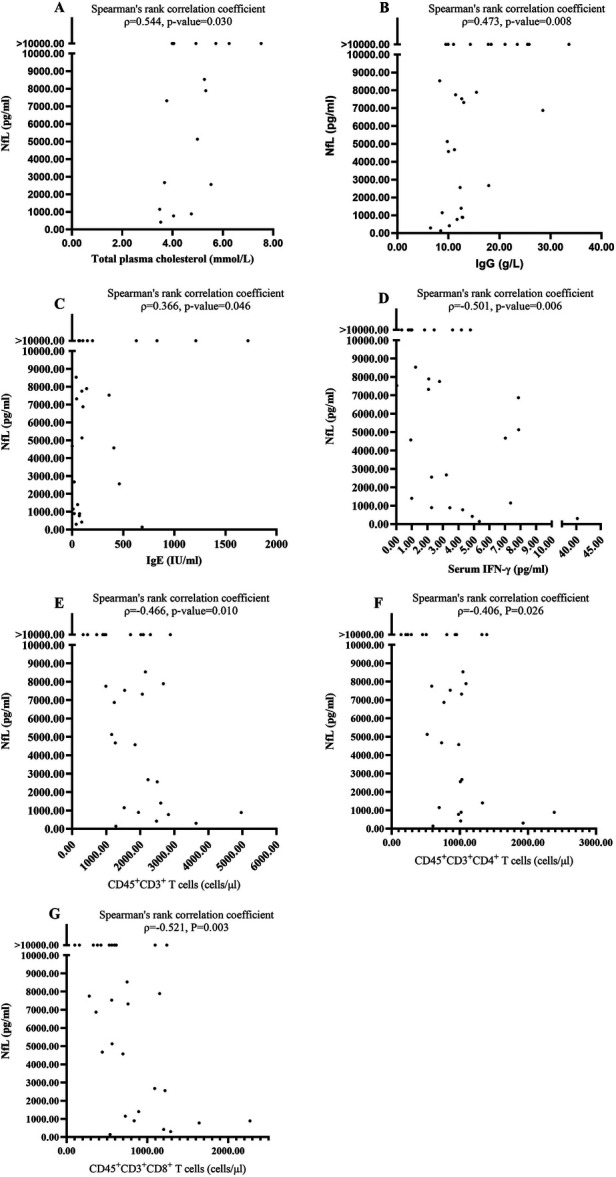
The significant correlations between NfL and laboratory results. **(A)** The CSF NfL positively correlated with the total plasma cholesterol. **(B)** The CSF NfL positively correlated with Immunoglobulin IgG. **(C)** The CSF NfL positively correlated with IgE. **(D)** The CSF NfL negatively correlated with serum IFN-*γ*. **(E)** The CSF NfL negatively correlated with peripheral blood CD45 ^+^ CD3^+^ T. **(F)** The CSF NfL negatively correlated with peripheral blood CD45 ^+^ CD3 ^+^ CD4^+^ T cells. **(G)** The CSF NfL negatively correlated with peripheral blood CD45 ^+^ CD3 ^+^ CD8^+^ T cells.

We analyzed the association between the CSF NfL for the first sample and other laboratory examinations in blood and CSF. The CSF NfL positively correlated with the total plasma cholesterol (Spearman's rank correlation coefficient, *ρ* = 0.544, *P* = 0.030; Figure 2A). However, it did not correlate with blood white blood cell (WBC) count, C-reactive protein, erythrocyte sedimentation rate and plasma triglyceride, high-density lipoprotein, and low-density lipoprotein (Spearman's rank correlation coefficient, *P* > 0.05). The CSF NfL positively correlated with immunoglobulin (Ig) G (Spearman's rank correlation coefficient, *ρ* = 0.473, *P* = 0.008; Figure 2B) and IgE (Spearman's rank correlation coefficient, *ρ* = 0.366, *P* = 0.046; Figure 2C), but not correlated with IgA, IgM, and complement 3 and complement 5 (Spearman's rank correlation coefficient, *P* > 0.05). Moreover, the CSF NfL did not correlate with CSF WBC count and CSF protein (Spearman's rank correlation coefficient, *P* > 0.05). Furthermore, there was no significant difference between patients with oligoclonal bands positive and without (Mann–Whitney *U* test, *Z* = −0.332, *P* = 0.740). After analyzing the association between the CSF NfL and cytokines, including tumor necrosis factor β, interleukin (IL) 12p70, IL-1 β, IL-10, IL-6, tumor necrosis factor α, interferon γ (IFN-γ), IL-8, IL-4, IL-5, IL-17A, and IL-22 in both serum and CSF, we found the CSF NfL negatively correlated with IFN-γ (Spearman's rank correlation coefficient, *ρ* = −0.501, *P* = 0.006; Figure 2D) in serum. However, it did not correlate with the others (Spearman's rank correlation coefficient, *P* > 0.05). In addition, the CSF NfL negatively correlated with peripheral blood CD45 ^+^ CD3^+^ T (Spearman's rank correlation coefficient, *ρ* = −0.466, *P* = 0.010; Figure 2E), CD45 ^+^ CD3 ^+^ CD4^+^ T (Spearman's rank correlation coefficient, *ρ* = −0.406, *P* = 0.026; Figure 2F), and CD45 ^+^ CD3 ^+^ CD 8^+^ T cells (Spearman's rank correlation coefficient, *ρ* = −0.521, *P* = 0.003; Figure 2G). However, it did not correlate with CD45 ^+^ CD19^+^ B cells (Spearman's rank correlation coefficient, *P* = 0.782).

### 3.3 The association between NfL and MRI lesions

We analyzed the association between the CSF NfL for the first sample with MRI lesions. We found CSF NfL in patients with lesions in cerebral white matter was higher than that in patients without [median 8,535.76 pg/ml IQR (4,573.39, >10,000 pg/ml) vs. median 2,668.60 pg/ml IQR (885.65, 7,530.11 pg/ml), Mann–Whitney *U* test, *Z* = −2.008, *P* = 0.045). However, there was no significant difference between patients with or without in the other locations described above (Mann–Whitney *U* test, *P* > 0.05). Moreover, we found that the CSF NfL positively correlated with the number of MRI locations (Spearman correlation coefficient, *ρ* = 0.362, *P* = 0.049).

### 3.4 Longitudinal studies of NfL

Nine patients underwent multiple detections of CSF NfL with a total of 15 samples at follow-up (more details seen in Figure 1). At follow-up, 3 samples were taken at relapse attack from 2 patients with the interval from the first detection 1 month, 3 months, and 13 months, respectively (more details seen in Figure 1B). The other 12 samples were taken in remission at follow-up from 9 patients with the median interval from the first detection 1-month IQR (1, 4 months). Although the EDSS during remission at follow-up was significantly reduced compared to that at onset or during a relapse episode (Wilcoxon signed rank test, Z = −2.232, *P* = 0.026), there was no significant difference in CSF NfL levels between the last detection in remission and the initial detection (Wilcoxon signed rank test, Z = −0.526, *P* = 0.599). At follow-up, two patients underwent CSF NfL detection at relapse attack. The CSF NfL was lower than before in one patient (from 7,754.32 pg/ml to 5,215.08 pg/ml at the first relapse with 1-month interval; from 5,215.08 pg/ml to 1,969.57 pg/ml at the second relapse with 3 months interval), while unchanged in the other one (both were higher than 10,000 pg/ml before relapse and at relapse, attack with 8 months of interval).

## 4 Discussion

In this study, we analyzed the CSF NfL in children with ADS at onset attack, relapse attack, and follow-up and its associated factors of laboratory and imaging results. We found that the NfL increase over normal value was seen in 98.7% (29/30) of patients. CSF NfL positively correlated with the EDSS at onset or during a relapse episode. Moreover, there was no significant difference in CSF NfL for the first time between patients at onset and relapse attacks. Similar to the study by Boesen et al., they found CSF NfL was significantly higher in ADS than higher control or patients with non-demyelinating CNS disease without CNS inflammation or inflammatory neurological disease consisting of either inflammatory or infectious neurological disease or neurological diseases with CNS inflammation (2). NfL is a specific biomarker of neuro-axonal injury released into the CSF (6). Significantly increased NfL in ADS suggests providing evidence of axonal injury predominantly in ADS.

In healthy individuals, CSF NfL linearly correlated with age, and the exponential yearly increase in CSF NfL was around 3.1%, and CSF NfL was higher in healthy males compared to females (19, 20). However, we failed to find an association between CSF NfL and sex and age. That may be caused by the sample size limitation in our study. Moreover, we found no significant difference in CSF NfL between patients with different diagnoses, including uncategorized ADS, ADEM, MOGAD, and MS/NMO/NMOSD. In a study about CSF NfL in CNS inflammatory demyelinating disease, no statistically significant differences were found in the CSF NfL levels among the clinical isolated syndrome, MS, and NMOSD groups (21). It suggested that the axonal damage was similar between these diseases.

ADS comprises a spectrum of monophasic and recurrent inflammatory conditions of the central nervous system. So we analyzed the association of CSF NfL with the serum and neuroinflammatory markers. The CSF NfL did not correlate with CSF WBC count and CSF protein. Interestingly, we found the CSF NfL negatively correlated with serum IFN-γ, CD45 ^+^ CD3^+^ T, CD45 ^+^ CD3 ^+^ CD4^+^ T, and CD45 ^+^ CD3 ^+^ CD8^+^ T cells. NfL was released into the CSF and serum following neuro-axonal damage. Moreover, serum and CSF NfL concentrations are highly correlated (6). Serum NfL may act as self-antigens and activate T cells. These self-activated T cells underwent transient clonal expansion, followed by rapid death mediated by regulatory T cells, and IFN-γ production can be inhibited by a regulatory T cell (22–24).

We analyzed the association between the CSF NfL for the first time with MRI lesions. We found that CSF NfL in patients with lesions in cerebral white matter was higher than that in patients without and the CSF NfL positively correlated with the number of MRI locations. Similar to previous studies in MS, NfL levels correlate with MRI tissue damage and are associated with the number of cerebral and spinal MRI lesions (5, 25–27). These suggest NfL can be a biomarker of neuro-axonal injury and a good indication of the extent of lesions.

Longitudinal studies of NfL levels in NMOSD and MS show NfL increases during an acute attack and slowly decreases over time or normalizes after treatment (27, 28). Moreover, NfL is a useful biomarker to evaluate the treatment response of new medicines in MS patients (6, 29, 30). In our study, the CSF NfL for the last detection at follow-up was not significantly different from the first, with the median interval from the first detection 1 month IQR (1, 4 months). We failed to found the change of NfL from the first to the last detection may be caused by the sample limitation and the short detection interval. NfL levels increase steadily for 1–3 weeks after onset until they reach a plateau and then gradually decrease for 3–6 months after the insult (5, 31). When using NfL as disease activity monitoring, the half-life should be a key consideration for the frequency of samples taken.

Our study has several limitations. The study may have a small sample size, which can limit the generalizability of the findings to a larger population. Other factors such as age, sampling timepoint, and concurrent comorbidities or infections that may potentially influence the CSF NfL value were not accounted for or controlled in the analysis (1). The use of serum NfL percentiles and *Z* scores from a large reference database from a general population allows the quantitative assessment of deviation from normal NfL concentrations. If similar percentiles and *Z* scores were available for cerebrospinal fluid NfL, it could reduce the impact of confounding factors (2).

## 5 Conclusions

The CSF NfL increases significantly in pediatric ADS, and it can be a biomarker of neuro-axonal injury and a good indication of the extent of lesions.

## Data Availability

The original contributions presented in the study are included in the article/Supplementary Material; further inquiries can be directed to the corresponding author.

## References

[1] Rostasy K Bajer-Kornek B Venkateswaran S Hemingway C Tardieu M. Differential diagnosis and evaluation in pediatric inflammatory demyelinating disorders. Neurology. (2016) 87(9 Suppl 2):S28–37. 10.1212/wnl.0000000000002878 27572858

[2] Boesen MS Jensen PEH Magyari M Born AP Uldall PV Blinkenberg M Increased cerebrospinal fluid chitinase 3-like 1 and neurofilament light chain in pediatric acquired demyelinating syndromes. Mult Scler Relat Disord. (2018) 24:175–83. 10.1016/j.msard.2018.05.017 30055504

[3] Wynford-Thomas R Jacob A Tomassini V. Neurological update: MOG antibody disease. J Neurol. (2019) 266(5):1280–6. 10.1007/s00415-018-9122-2 30569382 PMC6469662

[4] Ferilli MAN Valeriani M Papi C Papetti L Ruscitto C Figà Talamanca L Clinical and neuroimaging characteristics of MOG autoimmunity in children with acquired demyelinating syndromes. Mult Scler Relat Disord. (2021) 50:102837. 10.1016/j.msard.2021.102837 33636614

[5] Thebault S Booth RA Freedman MS. Blood neurofilament light chain: the neurologist’s troponin? Biomedicines. (2020) 8(11):523. 10.3390/biomedicines8110523 33233404 PMC7700209

[6] Ziemssen T Arnold DL Alvarez E Cross AH Willi R Li B Prognostic value of serum neurofilament light chain for disease activity and worsening in patients with relapsing multiple sclerosis: results from the phase 3 ASCLEPIOS I and II trials. Front Immunol. (2022) 13:852563. 10.3389/fimmu.2022.852563 35432382 PMC9009385

[7] Giovannoni G. Peripheral blood neurofilament light chain levels: the neurologist’s C-reactive protein? Brain. (2018) 141(8):2235–7. 10.1093/brain/awy200 30060019

[8] Abdelhak A Barba L Romoli M Benkert P Conversi F D'Anna L Prognostic performance of blood neurofilament light chain protein in hospitalized COVID-19 patients without major central nervous system manifestations: an individual participant data meta-analysis. J Neurol. (2023) 270(7):3315–28. 10.1007/s00415-023-11768-1 37184659 PMC10183689

[9] Capasso N Virgilio E Covelli A Giovannini B Foschi M Montini F Aging in multiple sclerosis: from childhood to old age, etiopathogenesis, and unmet needs: a narrative review. Front Neurol. (2023) 14:1207617. 10.3389/fneur.2023.1207617 37332984 PMC10272733

[10] Jarius S Paul F Aktas O Asgari N Dale RC de Seze J MOG Encephalomyelitis: international recommendations on diagnosis and antibody testing. J Neuroinflammation. (2018) 15(1):134. 10.1186/s12974-018-1144-2 29724224 PMC5932838

[11] Krupp LB Tardieu M Amato MP Banwell B Chitnis T Dale RC International Pediatric Multiple Sclerosis Study Group criteria for pediatric multiple sclerosis and immune-mediated central nervous system demyelinating disorders: revisions to the 2007 definitions. Multiple sclerosis (Houndmills, Basingstoke. England. (2013) 19(10):1261–7. 10.1177/1352458513484547 23572237

[12] Wingerchuk DM Banwell B Bennett JL Cabre P Carroll W Chitnis T International consensus diagnostic criteria for neuromyelitis optica spectrum disorders. Neurology. (2015) 85(2):177–89. 10.1212/WNL.0000000000001729 26092914 PMC4515040

[13] Thompson AJ Banwell BL Barkhof F Carroll WM Coetzee T Comi G Diagnosis of multiple sclerosis: 2017 revisions of the McDonald criteria. Lancet Neurol. (2018) 17(2):162–73. 10.1016/S1474-4422(17)30470-2 29275977

[14] Armangue T Olivé-Cirera G Martínez-Hernandez E Sepulveda M Ruiz-Garcia R Muñoz-Batista M Associations of paediatric demyelinating and encephalitic syndromes with myelin oligodendrocyte glycoprotein antibodies: a multicentre observational study. Lancet Neurol. (2020) 19(3):234–46. 10.1016/S1474-4422(19)30488-0 32057303

[15] Hou C Wu W Tian Y Zhang Y Zhu H Zeng Y Clinical analysis of anti-NMDAR encephalitis combined with MOG antibody in children. Mult Scler Relat Disord. (2020) 42:102018. 10.1016/j.msard.2020.102018 32234601

[16] Chen L Chen C Zhong X Sun X Zhu H Li X Different features between pediatric-onset and adult-onset patients who are seropositive for MOG-IgG: a multicenter study in south China. J Neuroimmunol. (2018) 321:83–91. 10.1016/j.jneuroim.2018.05.014 29957392

[17] Zheng R Li Y Chen D Su J Han N Chen H Changes of host immunity mediated by IFN-*γ*(+) CD8(+) T cells in children with adenovirus pneumonia in different severity of illness. Viruses. (2021) 13(12):2384. 10.3390/v13122384 34960654 PMC8708941

[18] Zhao MQ Wang LH Lian GW Lin ZF Li YH Guo M Characterization of lymphocyte subsets in peripheral blood cells of children with EV71 infection. J Microbiol Immunol Infect. (2020) 53(5):705–14. 10.1016/j.jmii.2019.03.001 30914258

[19] Yilmaz A Blennow K Hagberg L Nilsson S Price RW Schouten J Neurofilament light chain protein as a marker of neuronal injury: review of its use in HIV-1 infection and reference values for HIV-negative controls. Expert Rev Mol Diagn. (2017) 17(8):761–70. 10.1080/14737159.2017.1341313 28598205

[20] Bridel C van Wieringen WN Zetterberg H Tijms BM Teunissen CE Alvarez-Cermeño JC Diagnostic value of cerebrospinal fluid neurofilament light protein in neurology: a systematic review and meta-analysis. JAMA Neurol. (2019) 76(9):1035–48. 10.1001/jamaneurol.2019.1534 31206160 PMC6580449

[21] Peng L Bi C Xia D Mao L Qian H. Increased cerebrospinal fluid neurofilament light chain in central nervous system inflammatory demyelinating disease. Mult Scler Relat Disord. (2019) 30:123–8. 10.1016/j.msard.2019.02.009 30771578

[22] Silber E Semra YK Gregson NA Sharief MK. Patients with progressive multiple sclerosis have elevated antibodies to neurofilament subunit. Neurology. (2002) 58(9):1372–81. 10.1212/WNL.58.9.1372 12011283

[23] Wong HS Park K Gola A Baptista AP Miller CH Deep D A local regulatory T cell feedback circuit maintains immune homeostasis by pruning self-activated T cells. Cell. (2021) 184(15):3981–97.e22. 10.1016/j.cell.2021.05.028 34157301 PMC8390950

[24] Panduro M Benoist C Mathis D. T(reg) cells limit IFN-*γ* production to control macrophage accrual and phenotype during skeletal muscle regeneration. Proc Natl Acad Sci U S A. (2018) 115(11):E2585–e93. 10.1073/pnas.1800618115 29476012 PMC5856564

[25] Wendel E-M Bertolini A Kousoulos L Rauchenzauner M Schanda K Wegener-Panzer A Serum neurofilament light-chain levels in children with monophasic myelin oligodendrocyte glycoprotein-associated disease, multiple sclerosis, and other acquired demyelinating syndrome. Mult Scler. (2022) 28(10):1553–1561. 10.1177/13524585221081090 35282740

[26] Yuan A Nixon RA. Neurofilament proteins as biomarkers to monitor neurological diseases and the efficacy of therapies. Front Neurosci. (2021) 15:689938. 10.3389/fnins.2021.689938 34646114 PMC8503617

[27] Dinoto A Sechi E Flanagan EP Ferrari S Solla P Mariotto S Serum and cerebrospinal fluid biomarkers in neuromyelitis Optica Spectrum disorder and myelin oligodendrocyte glycoprotein associated disease. Front Neurol. (2022) 13:866824. 10.3389/fneur.2022.866824 35401423 PMC8983882

[28] Hyun JW Kim SY Kim Y Park NY Kim KH Kim SH Absence of attack-independent neuroaxonal injury in MOG antibody-associated disease: longitudinal assessment of serum neurofilament light chain. Mult Scler. (2022) 28(6):993–9. 10.1177/13524585211063756 34965770

[29] Margoni M Preziosa P Tortorella P Filippi M Rocca MA. Does ocrelizumab limit multiple sclerosis progression? Current evidence from clinical, MRI, and fluid biomarkers. Neurotherapeutics. (2022) 19:1216–28. 10.1007/s13311-022-01252-5 35668317 PMC9587174

[30] Gärtner J Hauser SL Bar-Or A Montalban X Cohen JA Cross AH Efficacy and safety of ofatumumab in recently diagnosed, treatment-naive patients with multiple sclerosis: results from ASCLEPIOS I and II. Mult Scler. (2022) 28(10):1562–75. 10.1177/13524585221078825 35266417 PMC9315184

[31] Mariotto S Sechi E Ferrari S. Serum neurofilament light chain studies in neurological disorders, hints for interpretation. J Neurol Sci. (2020) 416:116986. 10.1016/j.jns.2020.116986 32563076

